# Factors Associated with patient satisfaction towards a prison detention Clinic Care among male drug-using inmates

**DOI:** 10.1186/s12913-022-08609-8

**Published:** 2022-10-17

**Authors:** Fang-Chun Hsieh, Lan-Ping Lin, Te-Pin Wu, Shang-Wei Hsu, Chao-Ying Lai, Jin-Ding Lin

**Affiliations:** 1 Civilian Division, Tri-Service General Hospital, National Defense Medical Center, Taipei, Taiwan; 2 School of Public Health, National Defense Medical Center, Taipei, Taiwan; 3 Institute of Long-Term Care, MacKay Medical College, No.46, Sec. 3, Zhongzheng Rd., Sanzhi District, 252 New Taipei City, Taiwan; 4 Sanitation and Health Section, Sindian Drug Abuser Treatment Center, Agency of Corrections, Ministry of Justice, New Taipei City, Taiwan; 5 Department of Healthcare Administration, Asia University, Taichung, Taiwan

**Keywords:** Patient satisfaction, Detention clinic care, Prison, Drug inmate, PSQ-18

## Abstract

This study assessed patient satisfaction and its associated factors among male drug-using inmates utilizing a prison detention clinic in Taiwan. A cross-sectional design and structured questionnaire were employed to recruit 580 drug-using inmates into the study. The Patient Satisfaction Questionnaire Short Form (PSQ-18), developed by the RAND Corporation, was used as the basis for the short scale of patient satisfaction, and the research data were analyzed using the SPSS for Windows 20.0 statistical software package. The results showed that the research subjects had low patient satisfaction in all the factors assessed compared with the scale’s general norms. Among the original seven satisfaction subscales in this study, the highest score was for the financial aspects, and the lowest was for the amount of time spent with doctors. This study also investigated satisfaction with medical lab exams and the pharmacy at the prison’s clinic, and the satisfaction scores were higher than the original seven subscales. In multiple logistic regression analyses, the final model indicated that the inmates undergoing observed rehabilitation (OR = 13.837, 95% CI = 2.736–69.983) were more likely satisfied with prison detention clinic c than those serving prison sentences. Those inmates with custodial deposits (high vs. low; OR = 1.813, 95% CI = 1.038–3.168), and meet their physical health needs (met vs. unmet; OR = 4.872, 95% CI = 2.054–11.560) had significant correlated with detention clinic care satisfactory level. Although there is only one study setting cannot give a generalizability for people who are incarcerated in Taiwan, this study highlights that the prison authorities should scrutinize factors associated with detention clinic care satisfaction, such as the type of inmate, economic status in the prison, self-reported health status, and their physical health needs, to increase the level of patient satisfaction.

## Introduction

Worldwide prison population is growing at a rate that exceeds the rate of population growth [1], it is estimated 11 million people are incarcerated in correctional facilities, and health-care provision in correctional systems is challenging [2]. Prisons are not healthy places, the prisoners have a high prevalence of communicable diseases, noncommunicable diseases, and mental health problems, and many unhealthy conditions, such as overcrowding and poor hygiene, are common [3]. In Australia, prison inmates have a history of high levels of drug use prior to imprisonment [4]. Almost two in three prison entrants reported using illicit drugs in the prior year [5]. In general, the health of prison inmates tends to be worse than in the general population, particularly among those who use drugs [6].

According to Winkelman et al. [7] reviewed the universal health coverage and incarceration, they concluded that the health care in prisons was often below the community standard and was independent of health coverage and financing in the general population. In the USA and Australia, where individuals in prison are excluded from public health insurance programmes [7–9]. In USA, there is a great challenge for the delivery of health care for incarcerated individuals is that many correctional systems require high co-payments for health care which will be a barrier for accessibility [2]. In Norway, a social health care system which they enforce the universal public insurance and restorative justice principles for the prisoners [9].

Despite the importance of prison health care, little is known about how prison health services and governance in the previous studies [9]. Generally, the data of healthcare for the prisoners is inadequate, even in high-income countries, prison health information systems are often archaic in nature, relying on paper-based records and needed to restructure [7]. However, as the United Nations Basic Principles for the Treatment of Prisoners [10] indicate that: “Prisoners shall have access to the health services available in the country without discrimination on the grounds of their legal situation” (Principle 9). The European Prison Rules also states that the general principle is that prisoners should enjoy an equivalent standard of care to persons outside prisons [11]. Good prison healthcare governance is essential for addressing health inequity, improving the health of broader communities and improving health care in prisons [12]. Therefore, it is necessary to understand the healthcare provision for this group of people, to insure they have the same right to health care as everyone else.

According to the World Prison Brief data [13], total prison population (including pre-trial detainees / remand prisoners) was 54,448, and prison population rate (per 100,000 of national population) was 231 in June, 2022. There were 49 correction establishments / institutions which included 24 prisons, 12 detention houses, 2 juvenile detention and classification houses, 2 juvenile reformatory schools, 2 juvenile correctional-educational schools, 3 vocational training institutes and 4 drug abstention and treatment centers. The Taiwan Prison Act [14], the supervisory authority of prisons is the Agency of Corrections, Ministry of Justice, and the law stimulates that “Prisons should monitor the physical and mental conditions of inmates and manage their medical treatment, preventive healthcare, screening, prevention of communicable diseases, and food hygiene.” A prison may, based on its size, can set up medical unit to process disease treatment, preventive healthcare measures, screening, prevention of communicable diseases, and dietary sanitation for inmates.

Sometimes, prisoners do not receive the medical care they need and to which they are entitled to while they are incarcerated [15]. The Madrid Recommendation from the World Health Organization states that health protection in prisons is essential for public health and called for the support and encouragement of countries and organizations to develop a comprehensive health program within all prisons [16]. Therefore, reorienting health service provisions, such as approaches emphasizing the importance of the early identification of key symptoms and risk factors, for prisoners is vital for the improvement of their health [17].

Although routine in the community, patient satisfaction surveys are relatively rare in prison settings [18]. Satisfaction surveys can provide useful insight into the experiences and expectations of patients and provide information that can help focus on the areas of patient experience that greatly impact on health outcomes [19]. Medical achievements and National Health Insurance (NHI) have led Taiwan to have one of the most excellent health care systems in the world. In order to extend the coverage of the NHI system, prison inmates were included in the system in January 2013. When a prisoner in Taiwan suffers from a disease, the corrections agency shall comply with relevant laws and regulations to invite physicians to conduct a careful diagnosis and subsequent treatment within the institution, and the prisoner should have priority in seeking medical treatment in the correctional institution. There are more than 100 prisons’ clinics in correctional institutions under Taiwan’s current NHI scheme. Mainstreaming health care in prisons is a vital health care policy in correctional institutions that provides equal health care accessibility and availability to inmates as well as the general population. To protect the health rights of prison inmates, it is necessary to assess the effectiveness of and satisfaction with the NHI system for this group of people. Therefore, this study surveyed the profile of detention clinic care provision and patient satisfaction to examine their associated factors among drug-using inmates in a prison detention center in Taiwan.

## Methods

### Design and research participants

This study had a cross-sectional design. The study population included a total of 750 male drug-using inmates in a prison detention center to observe drug abuse, treatment, and prison rehabilitation in north Taiwan. The research subjects were drug-using inmates recruited from this detention center in November 2013, excluding a total of 65 inmates with court appearances, medical treatments, family interviews, foreigners who participated in technical training classes, and 100 drug-using inmates imprisoned within the prior two weeks. The remaining 585 drug inmates, including 137 undergoing rehabilitation, 133 undergoing drug addiction treatment, 307 who were already sentenced, and 8 under quarantine, were recruited for this study. This article presents a general survey of the study. Five inmates refused to provide informed consent; therefore, 580 questionnaires were analyzed.

### Research Ethics and Protection

This study was reviewed and approved by the Institutional Review Board of Tri-Service General Hospital, National Defense Medical Center (TSGHIRB approval number: 2-102-05-082). The questionnaire was completed anonymously to ensure the respondents’ privacy and that the data’s confidentiality was maintained in accordance with individual laws. It was stipulated that the questionnaire’s content did not contain case statements, only for overall statistical analysis, and did not affect any rights of the prison inmate, and written informed consent was obtained from all subjects.

### Research Tools

This study used a self-designed structural questionnaire as a survey tool. The questionnaire framework was based on the Andersen Behavioral Model and Access to Medical Care [20, 21] as the theoretical basis and assessed the inmates according to their tendencies (i.e., predisposing factors), abilities (i.e., enabling factors), and medical care utilization needs (i.e., need factors). The Andersen health care utilization model is a conceptual model aimed at demonstrating the factors that lead to the use of health services. In practice, this study adopted this model to understand how and why participants use detention clinic care services, to assess inequality in access to detention clinic care services, and to examine the determinants that allow for equitable access to care. To understand the research subjects’ satisfaction with the prison’s medical treatment, the Patient Satisfaction Questionnaire Short Form (PSQ-18) [22], developed by the RAND Corporation, was used as the basis of the short scale of patient satisfaction including aspects such as overall satisfaction, medical technology quality, interpersonal communication, communication, financial issues, accessibility, and convenience of consultation time. The PSQ-18’s internal consistency reliability and correlation was 0.9, the satisfaction degree used Likert’s five-point method of 1–5 points for a total of 90 points. In summary, the PSQ-18 scale is a concise, validated tool that may be applied to various settings as well as to compare interventions such as in primary care and the detention clinic care department. This study added two questions from a self-made questionnaire to ask respondents about their satisfaction with the prison’s medical lab examinations and pharmacy prescriptions; thus, the total medical satisfaction score was 100 points.

### Questionnaire validity and reliability

To improve the validity and relatability of this study, the expert surface validity method (*n* = 5) was used to improve the questionnaire’s suitability, adequality, and readability upon completion of the questionnaire’s content design. Cronbach’s α coefficient analysis was conducted using the SPSS statistical software’s reliability analysis. The overall questionnaire’s Cronbach’s α coefficient was 0.754.

### Data Processing Method

After checking and debugging, the data were decoded and entered into the Microsoft Excel 2007 software according to the decoding book’s compilation. The statistical data analysis was conducted based on the research purpose and assumptions with the SPSS for Windows 20.0 statistical package software. The main statistical methods included descriptive statistics and inferential statistics. Single-variable analysis via the statistical chi-squared test was used to explore whether there were significant correlations between basic demographic characteristics, health behaviors, economic status, health status, and detention care satisfaction. The multiple logistic regression method was used to explore the associated factors that affected satisfaction with detention clinic care utilization. The regression model uses the Andersen’s behavioral model as a simple guide to choose variables related to medical care accessibility – predisposing, enabling, need’s factors. All the results were considered statistically significant at *p* ≤ 0.05.

## Results

### Descriptive characteristics of the study participants

The subjects’ demographic characteristics are shown in Table 1. The subjects’ average age was 39.3 years old. A total of 43.4% of the respondents completed junior high school and 45.3% completed senior high school; 58.4% of the subjects were married. There were three types of inmates in the prison detention center in this study: 52.1% of the subjects were sentenced to prison, 24.8% were detained and under observation or rehabilitation, and 23.1% were undergoing drug addiction treatment. Most of the inmates’ families had an economic income below 40,000 New Taiwan dollars (NTD) (54.7%), and the inmates’ custodial deposits—for personal daily expenses that are not covered by the prison—were below 2000 NTD (51%). A total of 55.2% of the subjects reported that they had good health, and 6.9% self-assessed that they had poor health. Overall, 36.4% of the subjects were diagnosed with chronic diseases in the prior year, and 77.1% reported that the detention center met their physical health needs.

**Table 1:** Characteristics of the study participants (*N* = 580)

Variables	***n***	%	Mean ± SD (range)
Age (*n* = 576)			39.3 ± 10 (18–88)
< 45	428	74.3	
≥ 45	148	25.7	
Education level (*n* = 580)			
Primary school and less	33	5.7	
Junior high school	252	43.4	
Senior high school	263	45.3	
College and more	32	5.6	
Marital status (*n* = 579)			
Unmarried	338	58.4	
Married	241	41.6	
Inmate type (*n* = 580)			
Inmates serving prison sentences	302	52.1	
Inmates undergoing drug addiction treatment	134	23.1	
Inmates under observation or in rehab	144	24.8	
Family income (*n* = 580)			
< 40,000 NTD	317	54.7	
≥ 40,000 NTD	159	27.4	
Unknown	104	17.9	
Custodial deposit (*n* = 561)			3050 ± 3341 (0–30,000)
< 2000 NTD	286	51	
≥ 2000 NTD	275	49	
Self-reported health status (*n* = 580)			
Good	320	55.2	
Fair	220	37.9	
Poor	40	6.9	
Diagnosed chronic diseases (*n* = 579)			
No	368	63.6	
Yes	211	36.4	
Met their physical health needs (*n* = 576)			
Yes	444	77.1	
No	132	22.9	

SD: standard deviation; NTD: New Taiwan Dollar

### Detention care utilization and satisfaction of the study subjects

Table 2 presents data on the subjects’ detention clinic care utilization in the prior month. A total of 396 (68.3%) subjects had used detention clinic care, among which 344 (68.6%) used the family medicine department, followed by 70 (13.9%) using dentistry, and 46 (9.2%) using the psychiatric department. In general, 57% of the subjects attended detention clinic care visits once per month, and 43% had more than two visits per month, for an average of 16.5 detention clinic care visits per person per year (calculated by the total detention clinic care visits 801 times per month/total 580 inmates × 12 months).

**Table 2:** Participants’ detention clinic care utilization in the prior month

Variable	***n***	%	Mean ± SD (range)
Have ever used detention care (*n* = 580)			
No	184	31.7	
Yes	396	68.3	
Clinical department (*n* = 502) ^a^			
Family medicine	344	68.6	
Dental clinic	70	13.9	
Psychiatric clinic	46	9.2	
Guard medication outside prison	13	2.6	
Unknown	29	5.8	
Detention care visits monthly (*n* = 365)			1.38 ^b^ (1–20)
1 visit	208	57.0	
≧ 2 visits	157	43.0	

^a^ Multiple choice. ^b^ Calculated by 801 visits/580 inmates = 1.38, so the average number of annual visits per person was 16.5 (1.38 visits × 12 months).

Table 3 shows the detention clinic care satisfaction scoring data among the drug inmates based on the PSQ-18 subscale. The main results are as follows:

**Table 3:** Statistics for the PSQ-18 subscales and constituent items among the participants

Subscales and constituent items	Strongly disagree	Disagree	Uncertain	Agree	Strongly agree
General satisfaction (mean ± SD = 3.22 ± 0.8)					
The medical care I have been receiving is just about perfect.	10 (2.6%)	47 (12.1%)	122 (31.4%)	155 (40%)	54 (13.9%)
I am dissatisfied with some things about the medical care I receive. ^*^	18 (4.7%)	94 (24.5%)	144 (37.5%)	103 (26.8%)	25 (6.5%)
Technical quality					
I think my doctor’s office has everything needed to provide complete care.	9 (2.3%)	50 (12.91%)	120 (30.8%)	157 (40.4%)	53 (13.6%)
Sometimes doctors make me wonder if their diagnosis is correct. ^*^	41 (10.6%)	155 (39.9%)	102 (30.9%)	59 (15.2%)	13 (3.4%)
When I go for medical care, they are careful to check everything when treating and examining me.	8 (2.1%)	34 (8.8%)	108 (28%)	171 (44.3%)	65 (16.8%)
I have some doubts about the ability of the doctors who treat me. ^*^	19 (4.9%)	97 (25.2%)	151 (39.2%)	95 (24.7%)	23 (6%)
Interpersonal manner					
Doctors act too businesslike and impersonal toward me. ^*^	17 (4.4%)	68 (17.5%)	127 (32.7%)	140 36.1%)	36 (9.3%)
My doctors treat me in a very friendly and courteous manner.	9 (2.3%)	27 (7%)	95 (24.7%)	193 (50.2%)	61 (5.8%)
Communication					
Doctors are good about explaining the reason for medical tests.	12 (3.1%)	51 (13.1%)	89 (22.9%)	170 (43.7%)	67 (17.2%)
Doctors sometimes ignore what I tell them. ^*^	15 (3.9%)	80 (20.7%)	127 (32.9%)	138 (35.8%)	26 (6.7%)
Financial aspects					
I feel confident that I can get the medical care I need without being set back financially.	15 (3.9%)	42 (11%)	63 (16.5%)	183 (47.9%)	79 (20.7%)
I have to pay for more of my medical care than I can afford. ^*^	45 (11.6%)	107 (27.6%)	131 (33.8%)	88 (22.7%)	17 (4.4%)
Time spent with doctors					
Those who provide my medical care sometimes hurry too much when they treat me. ^*^	15 (3.9%)	70 (18%)	121 (31.2%)	149 (38.4%)	33 (8.5%)
Doctors usually spend plenty of time with me.	16 (4.2%)	54 (14%)	120 (31.2%)	149 (38.7%)	46 (11.9%)
Accessibility and convenience					
I have easy access to the medical specialists I need.	30 (7.7%)	73 (18.8%)	135 (34.8%)	117 (30.2%)	33 (8.5%)
Where I get medical care, people have to wait too long for emergency treatment. ^*^	14 (3.6%)	67 (17.3%)	121 (31.3%)	146 (37.7%)	39 (10.1%)
I find it hard to get an appointment for medical care right away. ^*^	24 (6.2%)	105 (27.1%)	129 (33.3%)	94 (24.3%)	35 (9%)
I am able to get medical care whenever I need it.	9 (2.3%)	35 (9%)	135 (34.8%)	165 (42.6%)	44 (11.3%)
Satisfied with the medical lab exam testing	12 (3.1%)	23 (5.9%)	135 (34.8%)	172 (44.3%)	46 (11.9%)
Satisfied with the pharmacy services	14 (3.6%)	19 (4.9%)	115 (29.6%)	189 (48.6%)	52 (13.4%)

^*^ Reverse question; all of the items were scored so that the high scores reflected satisfaction with medical care

1. The general satisfaction score was 3.22 ± 0.80 points. A total of 53.9% of the subjects agreed or strongly agreed that the detention clinic’s medical care was perfect, and 33.3% reported they agreed or strongly agreed that they were dissatisfied with the medical care that they received.
2. The technical quality satisfaction score was 3.18 ± 0.70 points. A total of 54% of the subjects agreed or strongly agreed that the prison’s detention center provided complete medical care, 18.6% agreed or strongly agreed that “sometimes doctors make me wonder if their diagnosis is correct”, and 61.1% agreed or strongly agreed that medical staff carefully assessed everything when treating and examining them. Overall, 30.1% of the subjects disagreed or strongly disagreed with the doctors’ ability to treat them.
3. The interpersonal manner satisfaction score was 3.20 ± 0.78. A total of 45.4% of the subjects agreed or strongly agreed that the doctors acted too businesslike and impersonal, and 56% agreed or strongly agreed that the doctors treated them in a very friendly and courteous manner.
4. The communication satisfaction score was 3.19 ± 0.82 points. A total of 60.9% of subjects agreed or strongly agreed that the doctors explained the diagnosis and examination, and 63 respondents (16.2%) disagreed. Overall, 42.5% of the subjects agreed or strongly agreed that the doctor ignored what they said.
5. The financial satisfaction score was 3.44 ± 0.76 points. A total of 262 subjects (68.6%) agreed or strongly agreed that they did not worry about obtaining medical care because of economic problems. However, 27.1% said that they had to pay for more medical care than they could afford.
6. The average satisfaction score for time spent with doctors was 3.05 ± 0.81 points. A total of 85 subjects (21.9%) agreed or strongly agreed that medical care was provided without rushing, and 182 (46.9%) disagreed or strongly disagreed. Overall, 50.6% of the subjects agreed or strongly agreed that the doctor was willing to take the time to talk to them in detail, and 70 participants (18.2%) disagreed or strongly disagreed.
7. The average satisfaction score for medical accessibility and convenience was 3.07 ± 0.62 points. A total of 150 subjects (38.7%) agreed or strongly agreed that it was easy to see a specialist, and 103 (26.5%) disagreed or strongly disagreed. Overall, 47.8% of the subjects agreed or strongly agreed that they had to wait too long for emergency treatment. A total of 33.3% of the subjects reported that it was difficult to make an appointment for immediate medical care, and only 11.3% reported that they were unable to make an appointment for immediate medical care.
8. The average satisfaction scores for medical lab testing and pharmacy services were 3.56 ± 0.88 points and 3.63 ± 0.90 points, respectively. The majority of the subjects agreed or strongly agreed that they were satisfied with the prison’s medical lab exams (52.2%) and pharmacy services (62%).

### Correlates of Detention Care satisfaction scores among the study subjects

Table 4 describes the distribution of the total detention clinic care satisfaction scores. Detention clinic care satisfaction was allocated 5 points for each question, and out of 20 questions, the total score was 100 points. A total of 5.6% of the subjects scored 26 to 50 points, 79.4% scored from 51 to 75 points, and 15% scored from 76 to 100 points, for an average of 64.55 ± 11.23 points. Using the 75th percentile as the cut-off point, satisfaction was divided into a high satisfaction group and a low–middle satisfaction group with 71 points. Overall, 89 subjects (25.1%) were in the high satisfaction group and 265 (74.9%) were in the low–middle satisfaction group.

**Table 4:** Satisfaction with detention clinic care among the participants (*n* = 354)

Scores and level	***n***	%	Mean ± SD (range)
Score distribution ^a^			64.55 ± 11.23 (29–100)
Level of satisfaction ^b^			
Moderate and low (< 71)	265	74.9	
High ( ≧ 71)	89	25.1	

^a^ Scoring: strongly disagree to strongly agree, score of 1–5. ^b^ Cut-off point for satisfactory level: 75th percentile

Analysis of the single variables of the subjects’ characteristics and detention care satisfaction (Table 5) showed that the type of inmate (*p* = 0.001), length of stay in the prison’s detention center (*p* = 0.001), custodial deposit (*p* = 0.025), and self-reported health status (*p* = 0.005) met their physical health needs (*p* = 0.002) and were significant factors related to the satisfactory level of detention clinic care utilization. The other variables, such as age, education, marital status, occupation, familial income, number of diseases, and diagnosed chronic diseases, were not significantly correlated with the satisfactory level of detention clinic care utilization.

**Table 5:** Univariate analyses of participants’ satisfaction towards detention clinic care (*n* = 354)

Variable	Moderate and low satisfaction	High satisfaction		
	***n*** **(%)**	***n*** **(%)**	**χ** ^**2**^	***p*** **-value**
Inmate type			25.958	0.001
Inmates serving prison sentences	156 (44.1)	35 (9.9)		
Inmates undergoing drug addiction treatment	75 (21.2)	21 (5.9)		
Inmates under observation or in rehab	34 (9.6)	33 (9.3)		
Length in prison’s detention center			15.938	0.001
< 2 months	49 (13.8)	34 (9.6)		
2–12 months	181 (51.1)	50 (14.1)		
> 12 months	35 (9.9)	5 (1.4)		
Custodial deposit (*n* = 342)			5.029	0.025
< 2000 NTD	134 (39.2)	33 (9.6)		
≥ 2000 NTD	122 (35.7)	53 (15.5)		
Self-reported health status			10.685	0.005
Good	117 (33.1)	56 (15.8)		
Fair	122 (34.5)	24 (6.8)		
Poor	26 (7.3)	9 (2.5)		
Satisfied with physical health needs			9.269	0.002
Yes	199 (56.2)	81 (22.9)		
No	66 (18.6)	8 (2.3)		

Non-significant variables: age, education, marital status, occupation, familial income, number of diseases, and diagnosed chronic diseases

In the multivariate analysis, the correlation between the subjects’ traits and detention clinic care satisfaction was analyzed using multiple logistic regression. As shown in Table 6, model 1 demonstrated that the inmates undergoing observed rehabilitation (OR = 8.638, 95% CI = 1.841–40.525) were more likely to be satisfied with detention clinic care than those serving prison sentences. Upon adding the economic status and health status of the research subjects in model 2, the research results showed that the type of inmate, custodial deposit, self-assessment of health status, and physical health needs were the factors that influenced detention care satisfaction. In multiple logistic regression analyses, the final model indicated that the inmates undergoing observed rehabilitation (OR = 13.837, 95% CI = 2.736–69.983) were more likely satisfied with prison detention clinic c than those serving prison sentences. Those inmates with custodial deposits (high vs. low; OR = 1.813, 95% CI = 1.038–3.168), and meet their physical health needs (met vs. unmet; OR = 4.872, 95% CI = 2.054–11.560) had significant correlated with detention clinic care satisfactory level.

**Table 6:** Multiple logistic regression analyses of participants’ levels of satisfaction towards detention clinic care (*n* = 354)

Variable (reference group)		Model 1		Model 2
	**β**	**OR (95% CI)**	**β**	**OR (95% CI)**
Constant	–2.198	0.111	-3.779	0.023
Inmate type (ref: those serving prison sentences)				
Inmates undergoing drug addiction treatment	0.045	1.046 (0.553–1.976)	-0.005	0.995 (0.507–1.951)
Inmates under observation	2.156	8.638 (1.841–40.525)^**^	2.627	13.837 (2.736–69.983)^**^
Length in prison’s detention center (ref: <2 months)				
3–12 months	0.881	2.413 (0.522–11.155)	1.173	3.233 (0.659–15.870)
> 12 months	0.253	1.287 (0.222–7.472)	0.575	1.777 (0.292–10.830)
Custodial deposit (ref: <2000 NTD)				
≥ 2000 NTD			0.595	1.813 (1.038–3.168)^*^
Self-reported health status (ref: poor)				
Fair			-0.868	0.420 (0.159–1.111)
Good			-0.090	0.914 (0.364–2.293)
Meet their physical health needs (ref: no)				
Yes			1.584	4.872 (2.054–11.560)^**^

^*^p < 0.05 and ^**^p < 0.01

## Discussion

Imprisonment affects the health and health needs of prisoners, and evidence-based prison health services can be provided for all inmates needing treatment, care, and prevention [23]. Stone et al. [24] stated that many correctional facilities, however, are not able to fully engage in continuous quality improvement activities mainly because of a lack of current, relevant quality models and benchmarks to serve as a basis for evaluation. However, PSQ-18 is an effective tool that is streamlined and suitable for various situations and comparative interventions [25], such as general practitioners [26], ophthalmology clinics [27], and psychiatry clinics [28], and is applied in different countries [29, 30]. To evaluate the impact of the prison’s primary medical care, the PSQ-18 short form scale was used to assess the subjects’ satisfaction with the prison’s medical service. The present study found that nearly 70% of the subjects used the prison’s clinic care, including 68.6% in family medicine, 13.9% in dentistry, and 9.2% in psychiatry. The results were generally consistent with other studies in Taiwan [31, 32], but the psychiatric care use was slightly lower.

This study revealed that most of the subjects came from low economic status families, consistent with another study [5] that concluded that those in contact with the criminal justice system had higher rates of homelessness and unemployment and often came from socioeconomically disadvantaged backgrounds. This study also reported that 36.4% of the subjects were diagnosed with chronic diseases in the prior year. The Australian statistics showed that almost one-half (45%) of female entrants had a history of chronic conditions, compared with almost 3 in 10 (28%) of male subjects [5]. The US Department of Justice also reported that prisoners and jail inmates were more likely than the general population to report ever having a chronic condition or an infectious disease [33].

Compared with the US patient satisfaction survey based on the PSQ-18 scale [22], the present results show that the inmates’ detention clinic care satisfaction was lower than the scale’s norms. Many previous studies have also indicated lower patient satisfaction among prison inmates. In a Norwegian study, prison inmates’ satisfaction with the health services provided were low compared with patient satisfaction in other health areas, particularly in the health service resources and quality of drug abuse treatment [34]. According to more than 100,000 inmates surveyed in the US, more than one-half were satisfied or very satisfied with the health care they received while incarcerated. In jails, 51% of the inmates surveyed reported being satisfied or very satisfied and in prisons it was 56% of those surveyed [33]. The results of a patient satisfaction survey conducted in the Connecticut prison system revealed that 43% of the inmates reported satisfaction with their health care [18]. In general, the prison group reported significantly lower satisfaction compared to the community group in illicit drug treatment [35].

The multiple logistic regression model in this survey indicates that service satisfaction provided by the detention care are associated with participant’s disposing factor - those will serve prison sentences, enabling factors such as those with custodial deposits (economic status), and the need factors such as the inmates undergoing drug addiction treatment, self-reported health status, and meet their physical health needs will also affect their perception to healthcare satisfaction. These factors needed to pay much attention in detention clinic to improve quality care for this group of people.

Taiwan’s NHI scheme may be said to be a high-performing health care system compared to many other health care systems around the world. Many characteristics contributing to the NHI’s high performance include the single payer system, used to set and regulate fees, and the imposition of a global budget system that caps total NHI expenditure [36]. The public’s satisfaction with the Taiwanese NHI has been high; it averaged around 80% in 2014 [37]. This study found that the highest satisfactory score was the financial aspects, and the lowest was time spent with doctors. Prison inmates have been included in Taiwan’s NHI system since January 2013. The problem associated with a short supply of medical services has since been greatly improved, and with the NHI’s financing mechanism and proper control and management, inmates, too, can have access to the reasonable use of health care resources [38]. General speaking, this health policy removed economic obstacles, making the subjects’ access to health care easier and, therefore, it was more satisfactory than the other factors. The other factor of limited health care consultation time in the prison illustrates the subjects’ demand for quality medical care and human rights protections. Thomson et al. [39] reported that health care provided in prisons was shifting from a basic level of care to a greater role in inmate health and identified prison inmates’ challenges and barriers to health care. The future top issues in prisons might be the confidentiality of medical information, standards of care, mental disorders and disabilities, and substance abuse and treatment [40].

Due to the specific needs of drug inmates, this study also assessed their satisfaction with the pharmacy services, and the results demonstrated that the satisfaction score was higher than the original seven subscales. The main reasons may be the high accessibility and affordability of these services, and one full-time pharmacist to provide services in the institution. The inmates can collect prescriptions immediately rather than waiting long for the medicines. There is good evidence to support the expanding role of pharmacists as primary care providers through activities such as direct patient care, health care clinics, and medication management in the corrections setting [41].

This study’s limitations were as follows: The survey subjects were required to sign an interview consent form. The subjects may have been concerned about their privacy and their own rights and interests, which could have affected the credibility of their answers to sensitive questions. As the questionnaire involved self-reported data, recall bias might also have occurred. There are many issues did not considered in this survey which might affect their healthcare needs and satisfaction, such as lacking of health conditions of the participants, undergoing withdrawal management and being sentenced to prison, key contextual factors etc. Although the PSQ-18 scale is an adaptable, reliable, and validated tool for use in various settings [42], including Asian countries such as Hong Kong [43], China [44], Malaysia [45], Thailand [46], and the Philippines [47]. There are a few studies that have been conducted in Taiwan that can be used as a comparison for this study. However, this study was one of the first to assess patient satisfaction among drug-using inmates in Taiwan, so the data can provide useful information for future health care policy initiatives to improve their quality of care.

## Conclusion

The results show that the subjects’ detention care satisfaction was lower than the scale’s original general norms; the subjects seemed more likely to be satisfied with the financial aspects and dissatisfied with the amount of time spent with doctors. However, this study only conduced in only one setting cannot give a generalizability for people who are incarcerated in Taiwan, furthermore, the medical care context between dentation center and the society are different in general. It is needed to conduct further investigation for assertion of their medical care satisfaction to this particular population. By using the Andersen health care utilization model as a guide to assess the medical care accessibility, the usage of detention care services is determined by three dynamics: predisposing, enabling, and need factors. This research highlights that prison authorities should scrutinize the factors associated with medical care satisfaction, such as the types of inmates (i.e., predisposing factor), economic status (enabling factor), and their physical health needs (i.e., need factors), to increase the level of patient satisfaction. Regarding future prison health policies, we suggest adapting the WHO’s European Network on Prison and Health [48], which states that managing and coordinating all relevant agencies and resources contributing to the health and well-being of prisoners is a whole-of-government responsibility and health authorities should provide and be accountable for health care services in prisons and advocate for healthy prison conditions.

## Data Availability

The data that support the findings of this study are available on request from the corresponding author. The data are not publicly available due to their containing information that could compromise the privacy of research participants.
